# Self-reported pregnancy-related health problems and self-rated health status in Rwandan women postpartum: a population-based cross-sectional study

**DOI:** 10.1186/s12884-016-1138-y

**Published:** 2016-11-07

**Authors:** Jean Paul S. Semasaka, Gunilla Krantz, Manasse Nzayirambaho, Cyprien Munyanshongore, Kristina Edvardsson, Ingrid Mogren

**Affiliations:** 1 Department of Clinical Sciences, Obstetrics and Gynecology, Umeå University, Umeå, Sweden; 2 University of Rwanda College of Medicine and Health Sciences School of Public Health, Kigali, Rwanda; 3 Department of Community Medicine and Public Health, Sahlgrenska Academy, University of Gothenburg, Gothenburg, Sweden; 4 Judith Lumley Centre, La Trobe University, Melbourne, Australia

**Keywords:** Self-rated health status, Pregnancy, Rwanda, Reproductive history, Cross-sectional study, Pregnancy-related complications

## Abstract

**Background:**

Self-rated health status (SRH) can be used as a predictor of morbidity and mortality. Postpartum self-rated health has been used to estimate maternal morbidity and postpartum problems. Reproductive history factors are associated with poor self-rated health postpartum. This study investigated prevalence of self-reported health problems during first, second, and third trimesters of pregnancy, delivery, and postpartum. In addition, this study investigated SRH in Rwandan women up to 13 months from partus.

**Methods:**

This population-based, cross-sectional study collected data in 2014 using structured interviews (*N* = 921). Univariable analysis was used to identify variables that were associated with poor self-rated health status (poor-SRH). Logistic regression analyses were performed to identify factors associated with poor-SRH at one day, one week, and one month postpartum and at the time of the interview.

**Results:**

Mean time between latest delivery and the time of interview was 7.1 months. Prevalence of anaemia, hypertension, diabetes mellitus during pregnancy, and severe bleeding during pregnancy and labour were 15.0, 4.9, 2.4, and 3.7 %, respectively. The prevalence of poor-SRH was 32.2 % at one day postpartum, 7.8 % at one month, and 11.7 % at time of the interview. Hypertension during pregnancy and significant postpartum haemorrhage were associated with poor-SRH at one day and one week postpartum. Severe bleeding during pregnancy and labour were associated with poor-SRH at one week and one month postpartum. Infection and anaemia during pregnancy were associated with poor-SRH at one month postpartum and at the time of interview. The Kaplan-Meier curves illustrate restitution of health for most women during the study period.

**Conclusions:**

This population-based study reports a high prevalence of poor SRH status among Rwandan women in the early postpartum period. Identified factors associated with poor-SRH were severe bleeding, hypertension, infection, and anaemia during pregnancy and postpartum haemorrhage. These factors may be prevented or reduced by providing more frequent and specific attention during pregnancy and by providing timely measures that address complications during delivery, including adequate postpartum care.

## Background

Self-rated health status (SRH) is recognised as a global measure of estimation of quality of life and a good predictor of morbidity and mortality [1–3]. SRH uses subjective questions, to ask how people perceive their health status. These questions summarize the participants’ health status, including physical, psychological, and social dimensions that are not easily accessible to an objective observer [4, 5].

In 2012, an estimated 213 million pregnancies occured in the world and more than 1.5 million of these pregnancies had pregnancy-related complications [6, 7]. The most common pregnancy-related complications are postpartum haemorrhage, hypertensive disorders (including pre-eclampia and eclampsia), sepsis, obstructive or prolonged labour, and spontaneous abortion [8]. Health problems such as headache, low back pain, anxiety, depression, urinary incontinence, and feacal incontinence may remain after childbirth [9–12]. There is poor documentation globally about the extent and the nature of women’s postpartum health status [13–16]. Poor self-rated health status has been found to be associated with reproductive history factors such as being a single mother, higher parity, delivery by caesarean section, and young age [10, 17, 18].

In 2013, an estimated 323,197 women gave birth in Rwanda, and 14.7 % of these women were delivered by caesarean section [19, 20]. Rwanda, like other low income countries, has made sincere efforts during the last decade to decrease maternal mortality and morbidity [21]. In Rwanda, from 2010 to 2015, the four recommended antenatal care (ANC) visits by the World Health Organization (WHO) increased from 35 to 44.3 %, with the incease number of women having the first ANC visit during the first trimester of pregnancy from 38 to 56.3 %. Skilled provider assistance during delivery increased from 69 to 91 %, and uptake of postnatal care from 18 to 43 %. Moreover, maternal mortality ratio was estimated to have decreased from 476 to 210 per 100 000 live births during the same period [21–23].

### Rationale of the study

There is insufficient information on rates of pregnancy-related complications in Rwanda. In addition, health status postpartum in Rwandan women is not well described. This study aims to fill the knowledge gap in this area and to serve as a base for policy-makers in their decision-making in order to improve maternal and foetal health and women’s health postpartum.

## Aims

In a population-based sample, this study investigated the self-rated overall health status in relation to reproductive history within 13 months from partus. Specific aims to investigate were: *i)* prevalence of self-reported health problems during first, second, and third trimesters of pregnancy, delivery, and postpartum *ii*) self-rated overall health status and its determinants at one day, one week, and one month postpartum and at the time of the interview.

This study is part of the Maternal Health Research Programme (MaTHeR) undertaken by the University of Rwanda in collaboration with University of Gothenburg and Umeå University in Sweden.

## Methods

### The study setting

The Rwandan health system is decentralised with the community level as the first level of maternal health service provision. There are three community health workers (CHWs) in each village including *Animatrice de Santé Maternelle* (ASM), a CHW who is exclusively in charge of maternal health services [24, 25]. The secondary level of maternal health services is the health center. The majority of women with uncomplicated pregnancies deliver at health centres, while complicated cases are referred to the district hospital level or to the referral hospitals according to the severity of the pregnancy-related problem [25].

This study was conducted in two locations in Rwanda: the city of Kigali and the Northern Province. Kigali includes urban, semi-urban, and rural areas with a total population of 1,135,428 and 1910 villages. The Northern Province includes mainly semi-urban and rural areas with a population of 1,729,927 and 2881 villages [26].

### Methodology of the study and study participants

#### Sampling

This study applied a cross-sectional study design. A sample size of 922 women was calculated based on the estimated prevalence of pregnancy-related hypertension (10 %) [8] with an absolute precision of 5 %, a 10 % possibility of non-responses, and a design effect of 1.5. A sampling frame prepared by the National Institute of Statistics of Rwanda (NISR) for the Rwandan general population and household census conducted in 2012 was used. This sampling frame is a complete list of villages covering the Northern Province and Kigali. From this sampling frame, 48 villages (1 %) were randomly selected. Eligible participants were women who gave birth within 13 months before data collection and who were identified with the assistance of the ASM. The final sample consisted of 921 as one contact did not participate, giving a response rate of 99.9 %.

#### Questionnaire

A questionnaire was developed by the research team that included questions about sociodemographic and psychosocial factors, pregnancies before the latest pregnancy, latest pregnancy, latest delivery, and postpartum situation. Sociodemographic background characteristics included age, marital status, and educational level. The majority of questions in the questionnaire were closed ended questions with a fixed number of response alternatives and Likert-type scale questions. For five questions in this study, the respondents were also given the possibility to give additional written comments. The questionnaire included questions about previous pregnancies as well as more detailed questions related to health problems during latest pregnancy, latest delivery, and postpartum period. Participants were also asked to report their SRH at one day postpartum, one week postpartum, one month postpartum, and at the time of the interview. First written in English, the questionnaire was translated into Kinyarwanda. Thereafter, the questionnaire was tested in a pilot study. The pilot study included 36 women from a village neighbouring a selected village of the study. All 36 questionnaires were completed, and apart from adjustments of the wording of some questions, no major revision of the questionnaire was needed after the pilot study.

#### Data collection procedures

All data were collected by a group of 12 female experienced interviewers (nurses, midwives, and clinical psychologists) through individual structured interviews to secure completeness of data. Before conducting the interviews, the interviewers participated in a five-day training. The data collection was performed between July 2014 and August 2014. At the end of each day, during the first three days of data collection, at least one participant per village was re-interviewed in order to check the completeness of the questionnaires and the accuracy of data collected. After the primary data entry, the information from 100 questionnaires, each including the 117 variables used in this study, were re-registered to check the accuracy of the first data entry. In total, 30 errors were detected which corresponds to an error rate of 0.25 % (30/11700). The erroneous data were thereafter corrected.

### Dependent variables

The participants retrospectively reported their *SRH* four times postpartum: one day, one week, one month, and at the time of the interview. There were five available response options: very good, good, neither good nor poor, poor, and very poor. In a sub-part of analysis, the variable was dichotomised into two categories labelled good health status (good-SRH) for those who rated their health as very good or good, and poor health status (poor-SRH) for those who rated their health status as very poor or poor or neither good nor poor.

### Independent variables

Sociodemographic and psychosocial variables were analyzed as independent variables. *Women’s age* was a continuous numerical variable that was divided into five age categories: less than 25 years, 25–29 years, 30–34 years, 35–39 years, and more than 40 years. *Marital status* included married, cohabiting, separated or divorced, widowed, and unmarried or single. *Age at marriage* was a continuous numerical variable that was categorized into less than 20 years, 21–30 years, and more than 30 years. *Woman’s education* was a combination of two primary variables: *having attended school* (yes or no) and *educational level. Educational level* included primary level not complete, primary level completed, vocational training, secondary level senior 1–4, secondary school senior 5–6, tertiary level, and a do not know option. The two variables were grouped into four categories: no education, completed primary level, completed secondary school or vocational training, and tertiary university level. *Woman’s occupation* included student, unskilled worker, skilled worker, civil servant, not employed, and other employment. *Place of delivery* included delivery at home, on the way to the health facility, at health post/dispensary, at the health centre, at district/provincial hospital, at referral hospital, at a private clinic, at any other health facility, and at any other place. *Mode of delivery* included delivered vaginally without instruments, vaginally with forceps, vaginally with vacuum extraction, planned caesarean section, and emergency caesarean section*. Health insurance* included the categories no health insurance, community health-based health insurance, public health insurance, and private health insurance. A new variable *handicapping complication* was created for women who reported either *fistula*, *urinary incontinence*, or *fecal incontinence. First trimester* was defined as the first three months of the pregnancy. *Second trimester* was defined as four to six months of pregnancy. *Third trimester* was defined as seven months or more. Variables about main health problems during pregnancy and delivery were for each variable collected for the first, second, and the third trimesters. Thereafter, each variable – *diabetes mellitus, anaemia*, and *infections* (composed essentially by urinary infection) during the first, the second, the third trimester – were combined to become *diabetes mellitus during pregnancy*, *anaemia during pregnancy*, and *infections during pregnancy. Significant vaginal blood loss within 24 h after delivery* and *significant vaginal blood loss within the first weeks after delivery* were combined into *significant blood loss after delivery.* Further categorization of variables are presented in first three tables.

### Statistical analysis

Prevalence rates were calculated for description of different variables related to pregnancy, delivery, and postpartum. The study identified factors related to poor-SRH postpartum using univariable logistic regression analysis. Variables that were statistically significantly associated with poor-SRH status were considered for the final logistic regression model. Finally, a multivariable logistic regression model was built that calculated odds ratios (OR) and their 95 % confidence intervals (CI). In the multivariable model, forward stepwise regression was used. All statistically significant variables in univariable analyses were entered one by one to identify factors that had a relationship with poor-SRH at one day, one week, and one month postpartum and at time of the interview, keeping in the final model only factors that were statistically significant (*p* < 0.05). All multivariable models included number of births, women’s age, mode of delivery, and marital status for theoretical reasons. A Kaplan-Meier analysis using the curve “One minus survival” with a log rank test was constructed to illustrate the time-dependent self-rated poor health status in relation to anaemia during pregnancy, women’s level of education, and significant postpartum haemorrhage. This analysis was done to illustrate improvement of health status in women who reported low level of education, anaemia during pregnancy, or significant postpartum haemorrhage compared to those without these factors during the follow-up period. These analyses only included women rating their health as poor-SRH at one day postpartum. Thus, the Kaplan-Meier analyses included a sub-category of 296 participants. The time end point for each participant was the time of the interview. All analyses were performed in SPSS version 22.

## Results

### Sociodemographic and reproductive history characteristics

A total of 921 women aged 15 to 46 years with a mean age of 27.9 years were enrolled in this study. The average period between the date of the interview and the date of the latest delivery was 7.1 months (range: 1.4–14.3 months). The distribution of proportions in relation to the postpartum follow-up time were 35.3 % for 1–5 months, 31.6 % for 5.1–9 months, and 33.1 % for 9.1–14.3 months. Frequencies and percentages of sociodemographic and reproductive history characteristics of participants are presented in Table 1. Married or cohabiting women constituted 84.1 % of participants, and single or unmarried women corresponded to 13.4 % of the sample. Primiparus mothers represented 30.7 % of the participants and multiparous women with more than four births represented 24.2 % of the participants. A large majority of participants (87.6 %) reported a normal vaginal delivery at a health facility, and 5.1 % reported delivery at home or on the way to a health facility.

**Table 1:** Socio-demographic and reproductive history background characteristics of participants

Variables	Mean age (years)	SD^a^
Women’s mean age	27.89	6.02
Women’s mean number of years of education	5.80	3.10
Husband’s mean age	32.55	7.74
Husband’s mean number of years of education mean	6.14	3.16
	N	%
Women’s age (years)	920	99.9
< 25	295	32.0
25–29	277	30.1
30–34	212	23.0
35–39	98	10.6
≥ 40	38	4.1
Marital status	920	99.9
Single or unmarried	123	13.4
Widow	13	1.4
Separated or divorced	10	1.1
Cohabiting	292	31.7
Married	482	52.4
Age at marriage (years)	804	87.3
< 20	339	36.8
21–30	447	48.5
> 30	18	2.0
Woman’s education	921	100
No education	97	10.5
Completed primary level	608	66.0
Secondary school and vocational training	178	19.3
Tertiary, university level	38	4.1
Woman’s occupation	920	99.9
Student	17	1.9
Non skilled worker	529	58.3
Skilled worker	41	4.5
Civil servant	61	6.7
Not employed	257	28.3
Other employment	3	0.3
Place of delivery	919	99.8
At home or on the way to the health facility	47	5.1
At public health facility	862	93.6
At private or any other health facility	10	1.1
Mode of delivery	913	99.1
Vaginal without instruments^b^	798	86.6
Vaginal with forceps	4	0.4
Vaginal with vacuum extraction	0	0
Planned caesarean section	33	3.6
Emergency caesarean section	78	8.5
Number of births	889	96.5
1	273	30.7
2	32	3.6
3	221	24.9
4	148	16.6
> 4	215	24.2
Discharge time (days)	899	97.6
< 3	474	52.7
3	273	30.4
4–7	119	13.2
> 7	33	3.7
Health insurance	920	99.9
No insurance	188	20.4
Community health based insurance	686	74.5
Public insurance (RAMA, MMI, MIS/UR)^c^ and other private	47	5.1
ANC visits	915	99.3
Yes	910	98.8
No	5	0.5
Number of ANC visits	915	99.3
1–3	497	54.3
≥ 4	418	45.7
Religion	919	99.8
Catholicism	428	46.6
Protestantism	334	36.3
Adventist	77	8.4
Islam	16	1.7
Other religion	58	6.3
No religion	6	0.7
Husband’s age group (years)	774	84.0
< 25	78	8.5
25–29	240	26.1
30–34	195	21.1
35–39	119	12.9
40–44	86	9.3
≥ 45	56	6.1
Husband’s education	791	85.0
No education	100	10.9
Completed primary level	527	57.3
Secondary school and vocational training	117	12.7
Tertiary, university level	32	3.5
Don’t know	15	1.6
Household income per month	914	99.2
< 17,500 RwF	258	28.2
17,500–35,000 RwF	240	26.3
> 35,000 RwF	416	45.5

^a^SD = Standard deviation

^b^Vaginal without instruments = normal delivery without forceps nor vacuum extraction

^c^RAMA = La Rwandaise d’Assurance Maladie, MMI = Military Medical Insurance, MIS/UR = Medical Insurance of University of Rwanda

### Health problems during pregnancy and delivery period

Prevalence of self-reported, pregnancy-related health problems during first, second, and third trimesters of pregnancy are presented in Table 2. Self-reported prevalence of anaemia, hypertension, diabetes mellitus, and severe bleeding during pregnancy were 15.0, 4.9, 2.2, and 2.0 %, respectively, and 15.7 % reported being transferred pre-delivery from a health center to a district hospital or to a referral hospital due to health problems. Abnormal foetal position was reported in 3.9 % of cases.

**Table 2:** Prevalence of self-reported pregnancy-related problems in 1st, 2nd, and 3rd trimesters^a^ of pregnancy, and the cumulative prevalence^b^

Variable	1st trimester		2nd trimester		3rd trimester		Cumulative prevalence	
	*n*	%	*n*	%	*n*	%	*n*	%
Hypertension	28	3	14	1.4	17	1.8	45	4.9
Convulsions	18	2	3	0.3	5	0.5	22	2.4
Diabetes mellitus	11	1.2	10	1.1	3	0.3	20	2.2
Bad smelling and vaginal discharge	41	4.5	47	5.1	29	3.1	85	9.2
Anaemia	66	7.2	81	8.8	58	6.3	138	15.0
Severe vaginal bleeding			14	1.5	6	0.7	18	2.0
Severe or continuous headache			59	6.4	46	5.0	72	7.8
Dimness or blurring vision			99	10.9	62	6.7	119	12.9
Abdominal pain and severe bleeding			16	1.7	5	0.5	177	19.2
Vomiting			105	11.4	44	4.8	117	12.7
Fever			26	1.3	9	1.0	29	3.1
Leaking of fluid from vagina			54	5.9	61	6.6	82	8.9
Swollen extremities			55	6.0	135	14.7	157	17
Preterm premature rupture of membranes			4	0.4	12	1.3	16	1.7
Diarrhoea			18	2.0	7	0.8	25	2.7
Baby not moving normally			9	1.0	5	0.5	12	1.3
Abdominal pain			93	10.1	93	10.1	177	19.2
Regular and painful uterine contractions			14	1.5	18	2.0	29	3.1
Infection			12	1.3	11	1.2	19	2.1
Preterm labour					29	3.1	29	3.1

^a^The first trimester represents the first three months of pregnancy, the second trimester represents four to six months of pregnancy, and the third trimester was defined as seven months or more

^b^The third trimester does not include events or complications during delivery

### Health problems during postpartum period

A total of 8.5 % of participants reported significant postpartum haemorrhage immediately after delivery or within 24 h, and 4.7 % reported significant postpartum haemorrhage during their first week postpartum. At the time of the interview, the prevalence of almost daily or weekly irritability, anxiety, and depression was 6.5 %, 22.6 %, and 14.8 %, respectively. A total of 2.0 % of the participants reported having developed vaginal fistula, 0.4 % reported fecal incontinence, 2.4 % reported urinary incontinence, and 3.3 % reported problems related to an episiotomy during most recent delivery. Generalized fatigue almost daily or weekly was reported by 17.3 %, severe headache by 21.7 %, and low back pain by 26.4 %.

### Self-rated overall health status postpartum

**Fig. 1:**
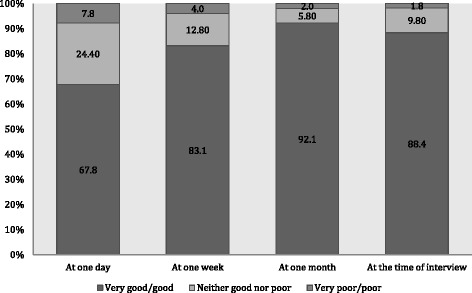
Self-rated health status postpartum at one day, one week, one month, and at time of interview. Different self-rated health status categories are presented with proportions (%)

**Fig. 2:**
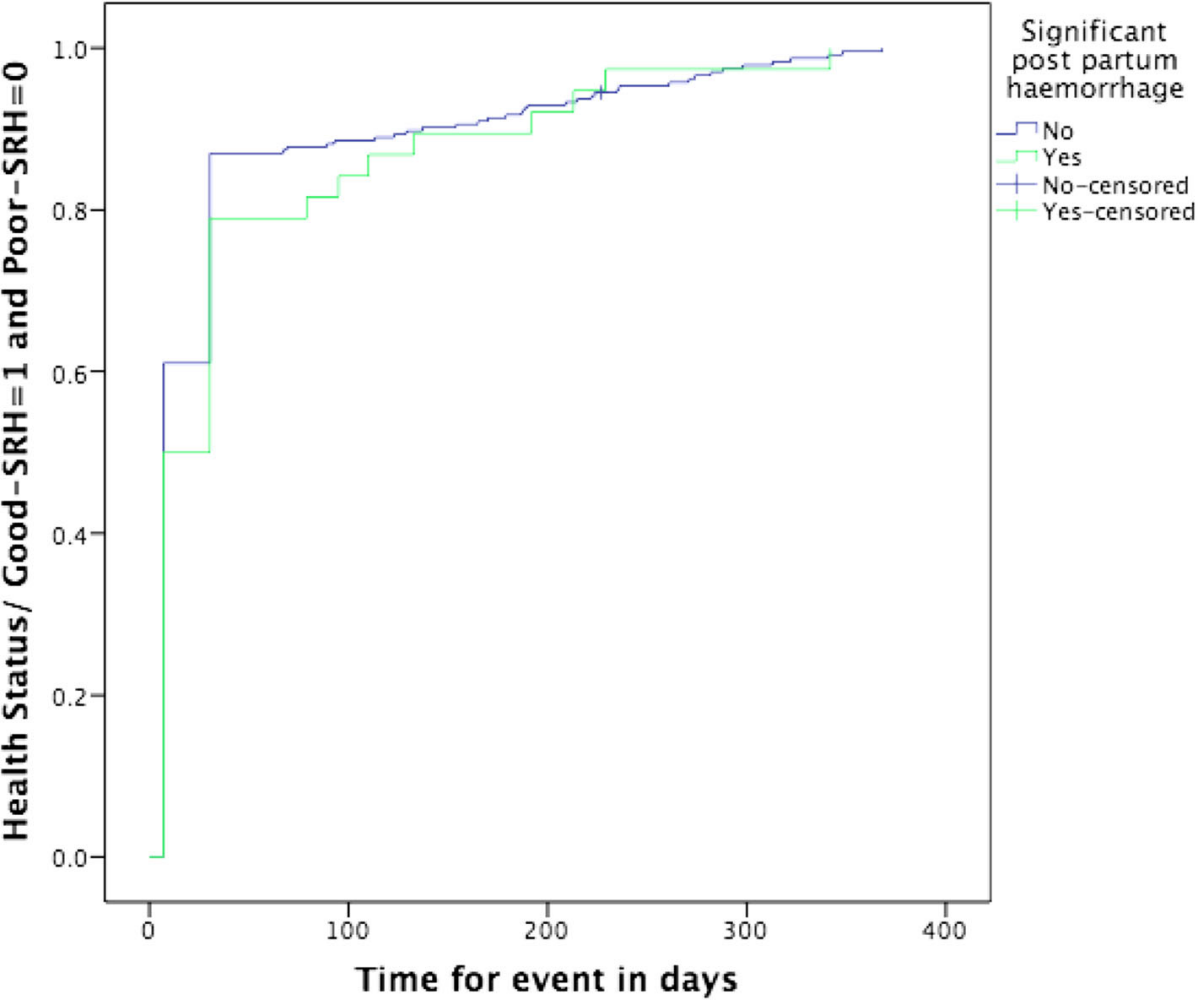
Kaplan-Meier curve showing change in health status from poor-SRH to good-SRH by experience of significant post partum haemorrage during follow-up time^a^ (*n* = 296^b^). ^a^Time of interview is time end point for follow-up of each woman. ^b^Only women reporting poor-SRH at day one are included in analysis

**Fig. 3:**
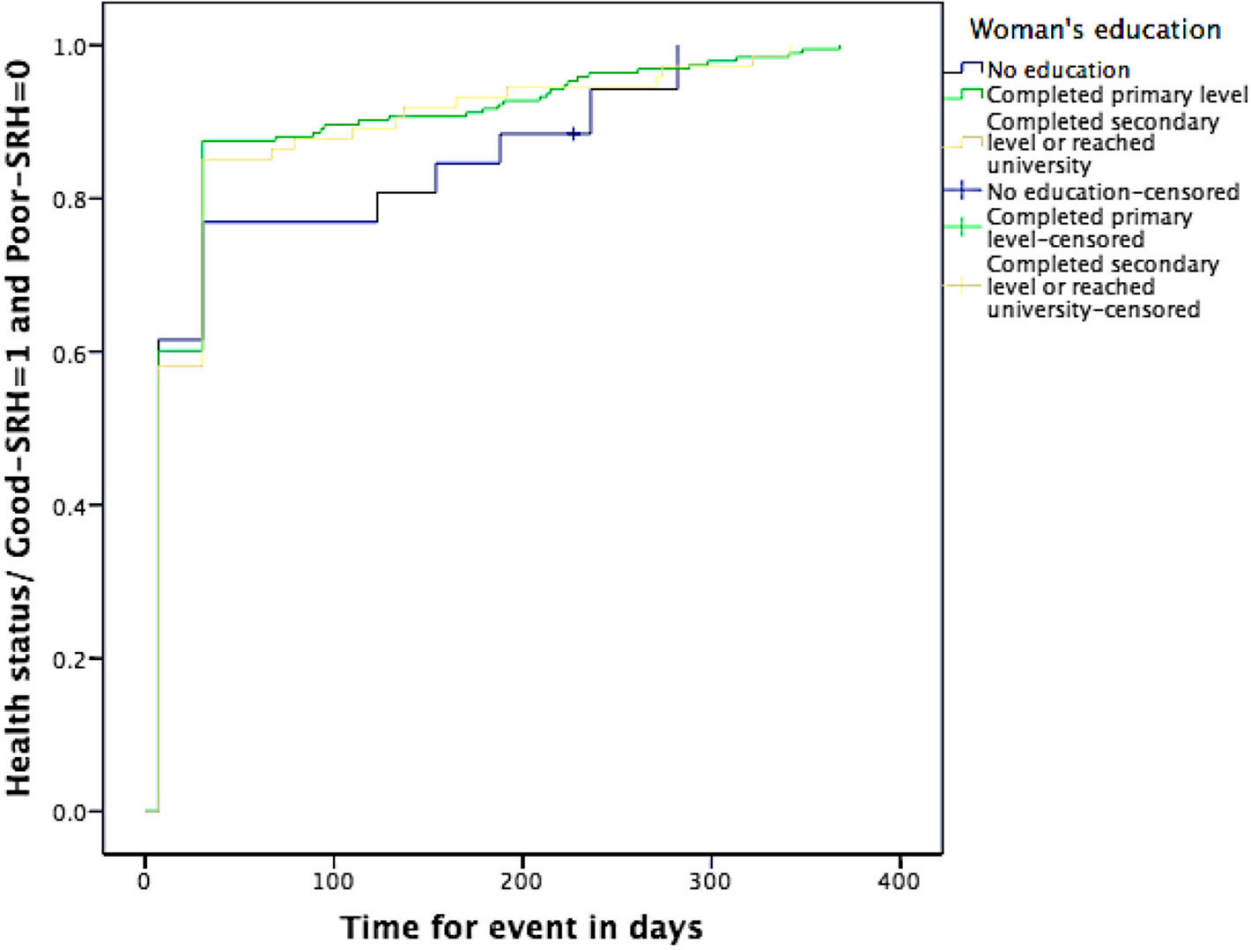
Kaplan-Meier curve showing change in health status from poor-SRH to good-SRH by educational level during follow-up time^a^ (*n* = 296^b^). ^a^Time of interview is time end point for follow-up of each woman. ^b^Only women reporting poor-SRH at day one are included in analysis

**Fig. 4:**
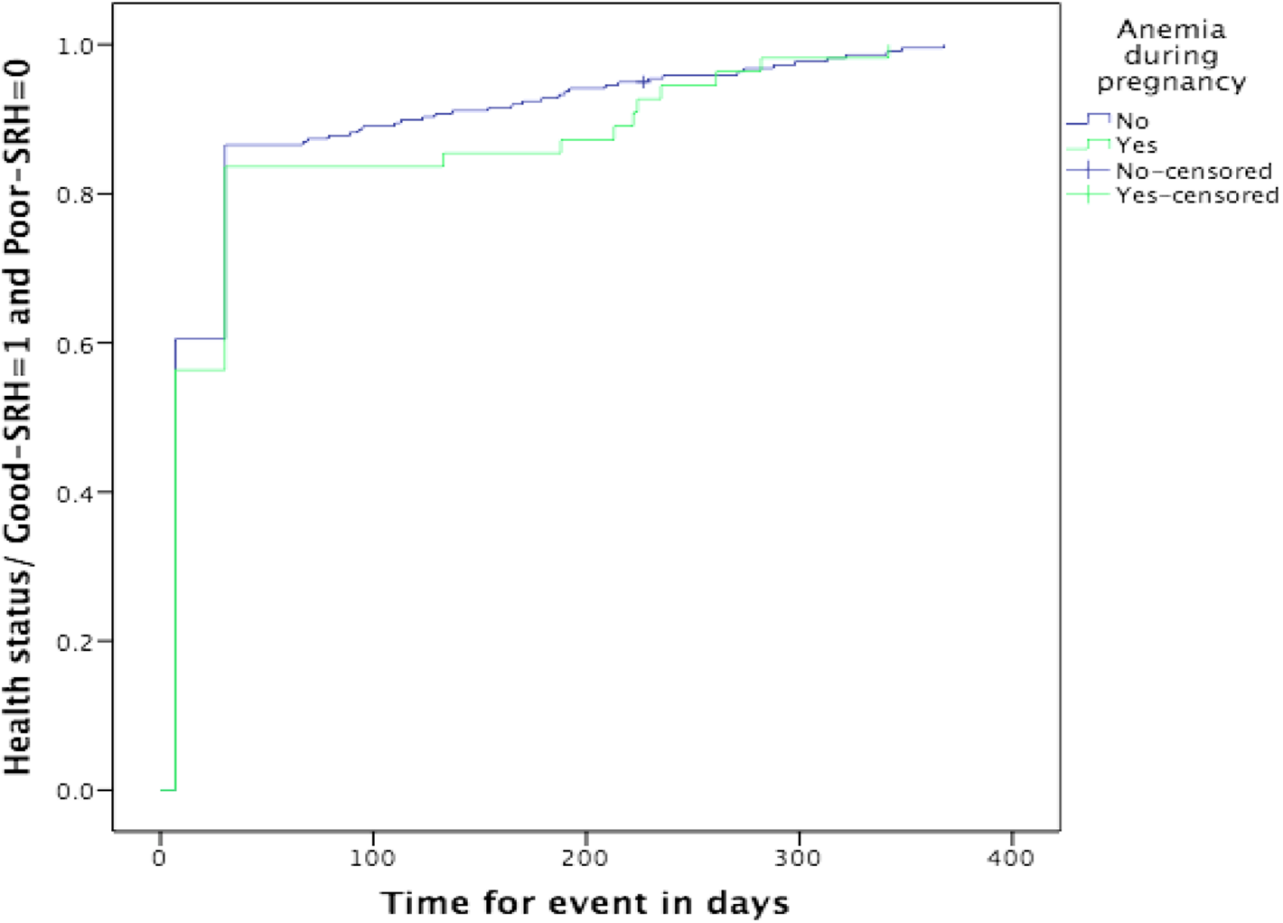
Kaplan-Meier curve showing change of health status from poor-SRH to good-SRH by anemia during pregnancy, during follow-up time^a^ (*n* = 296^b^). ^a^Time of interview is time end point for follow-up of each woman. ^b^Only women reporting poor-SRH at day one are included in analysis

The proportion of women who rated their overall health status as very poor or poor decreased during the study period (Fig. 1). Almost a third of participants (32.2 %) reported poor-SRH at one day, but only 11.7 % were still reporting poor-SRH at the time of the interview. The Kaplan-Meier curves illustrate that women with low level of education, women with anaemia during pregnancy, and women with significant postpartum haemorrhage reported poorer health status during the early part of the postpartum period, compared to women with none of these conditions. However, the log rank test revealed that for the entire follow-up period there was no statistically significant difference between groups for self-rated overall health status in relation to level of education (*p* = 0.863), anaemia during pregnancy (*p* = 0.463), and significant postpartum haemorrhage (*p* = 0.401). For those women who were followed-up to around one year postpartum, there was no significant difference in overall health status between women characterized by different categories of exposures (Figs. 2, 3, and 4). Thus, women had in general regained a good health status.

### Background characteristics and pregnancy outcomes and their associations with self-rated overall health status at different time points postpartum

#### Self-rated overall health status at one day postpartum

Reproductive factors that were associated with poor-SRH at one day postpartum in univariable logistic regression analyses are presented in Table 3. In a multivariable logistic regression model, the following reproductive factors were statistically significantly associated with poor-SRH at one day postpartum: caesarean section (reference group (ref.): vaginal delivery with or without instruments), hypertension during pregnancy and delivery (ref.: no hypertension), and significant postpartum haemorrage (ref.: no significant postpartum haemorrage) (Table 4).

**Table 3:** Good-SRH^a^ and poor-SRH^b^ in relation to background variables. Univariable logistic regression analysis^c^ for poor-SRH in relation to background variables at three times^d^

	SRH at day one after delivery			SRH at one month after delivery			SRH at the time of the interview		
	Good-SRH	Poor-SRH	Crude OR and its 95 % CI	Good-SRH	Poor-SRH	Crude OR and its 95 % CI	Good-SRH	Poor-SRH	Crude OR and its 95 % CI
Women’s age (years)									
< 25	198 (67.6 %)	97 (32.4 %)	1.02 (0.75–1.38)	265 (89.8 %)	30 (10.2 %)	**1.93 (1.12–3.32)** ^**e**^	263 (89.8 %)	30 (10.2 %)	0.82 (0.51–1.31)
25–34	329 (67.1 %)	158 (32.9 %)	1	461 (94.5 %)	27 (5.5 %)	1	427 (87.9 %)	59 (12.1 %)	1
≥ 35	96 (70.6 %)	40 (29.4 %)	0.86 (0.57–1.31)	122 (89.7 %)	14 (10.3 %)	1.95 (0.99–3.85)	118 (86.8 %)	18 (13.2 %)	1.10 (0.62–1.94)
Place of delivery									
At health facility	589 (67.5 %)	283 (32.5 %)	1	806 (92.4 %)	66 (7.6 %)	1	772 (89.0 %)	95 (11.0 %)	1
At home or on the way to health facility	33 (73.3 %)	12 (26.7 %)	0.75 (0.38–1.48)	40 (87.0 %)	6 (13.0 %)	1.83 (0.74–4.47)	36 (76.6 %)	11 (23.4 %)	**2.48 (1.22–5.04)**
Mode of delivery									
Vaginal (with or without instruments)	569 (71.1 %)	231 (28.9 %)	1	746 (93.1 %)	55 (6.9 %)	1	699 (87.7 %)	98 (12.3 %)	1
Caesarean Section	46 (41.4 %)	65 (58.6 %)	**3.48 (2.31–5.23)**	95 (85.6 %)	16 (14.4 %)	**2.28 (1.25–4.14)**	102 (91.9 %)	9 (8.1 %)	0.62 (0.30–1.28)
Hypertension during pregnancy and delivery									
No	602 (69.2 %)	268 (30.8 %)	1	808 (92.8 %)	63 (7.2 %)	1	767 (88.5 %)	100 (11.5 %)	1
Yes	21 (42.9 %)	28 (57.1 %)	**2.99 (1.67–5.37)**	40 (81.6 %)	9 (18.4 %)	**2.88 (1.34–6.21)**	42 (85.7 %)	7 (14.3 %)	1.27 (0.55–2.92)
Convulsions during pregnancy, delivery and post partum									
No	604 (68.3 %)	280 (31.7 %)	1	819 (92.5 %)	66 (7.5 %)	1	782 (88.7 %)	100 (11.3 %)	1
Yes	19 (54.3 %)	16 (45.7 %)	1.81 (0.92–3.58)	29 (82.9 %)	6 (17.1 %)	**2.56 (1.02–6.40)**	27 (79.4 %)	7 (20.6 %)	2.02 (0.86–4.77)
Diabetes mellitus during pregnancy									
No	608 (67.6 %)	291 (32.4 %)	1	828 (92.0 %)	72 (8.0 %)	1	791 (88.3 %)	105 (11.7 %)	1
Yes	15 (75.0 %)	5 (25.0 %)	0.69 (0.25–1.93)	20 (100.0 %)	0 (0.0 %)	0.000	18 (90.0 %)	2 (10.0 %)	0.83 (0.19–3.65)
Anaemia during pregnancy									
No	542 (69.4 %)	239 (30.6 %)	1	729 (93.2 %)	53 (6.8 %)	1	711 (91.4 %)	67 (8.6 %)	1
Yes	81 (58.7 %)	57 (41.3 %)	**1.59 (1.10–2.31**)	119 (86.2 %)	19 (13.8 %)	**2.19 (1.25–3.84)**	98 (71.0 %)	40 (29.0 %)	**4.33 (2.77–6.75)**
Marital status									
Married or cohabiting	514 (66.6 %)	258 (33.4 %)	1	712 (92.1 %)	61 (7.9 %)	1	687 (89.2 %)	83 (10.8 %)	1
Unmarried or single or widow or separated	108 (74.0 %)	38 (26.0 %)	**1.42 (0.95–2.12)**	135 (92.5 %)	11 (7.5 %)	1.05 (0.53–2.05)	121 (83.4 %)	24 (16.6 %)	**0.60 (0.37–0.99)**
Age at marriage (years)									
< 20	170 (69.7 %)	74 (30.3 %)	1	227 (93.0 %)	17 (7.0 %)	1	212 (86.9 %)	32 (13.1 %)	1
≥ 20	365 (65.4 %)	193 (34.6 %)	1.21 (0.87–1.67)	511 (91.4 %)	48 (8.6 %)	1.25 (0.70–2.22)	496 (89.2 %)	60 (10.8 %)	0.81 (0.50–1.26)
Number of births									
1	185 (67.8 %)	88 (32.2 %)	1	253 (92.7 %)	20 (7.3 %)	1	248 (91.2 %)	24 (8.8 %)	1
2–4	258 (64.7 %)	141 (35.3 %)	1.14 (0.82–1.59)	368 (92.0 %)	32 (8.0 %)	1.10 (0.61–1.96)	351 (88.2 %)	47 (11.8 %)	1.38 (0.82–2.32)
> 4	160 (74.4 %)	55 (25.6 %)	0.72 (0.48–1.07)	197 (91.6 %)	18 (8.4 %)	1.15 (0.59–2.24)	182 (84.7 %)	33 (15.3 %)	**1.87 (1.07–3.27)**
Discharge time (days)									
< 3	345 (72.8 %)	129 (27.2 %)	NA^f^	435 (91.8 %)	39 (8.2 %)	**1**	422 (89.4 %)	50 (10.6 %)	1
3	189 (69.2 %)	84 (30.8 %)		261 (95.6 %)	12 (4.4 %)	**0.51 (0.26–0.99)**	235 (86.4 %)	37 (13.6 %)	1.83 (0.42–7.90)
4–7	61 (51.3 %)	58 (48.7 %)		109 (91.6 %)	10 (8.4 %)	**1.02 (0.49–2.11)**	104 (88.9 %)	13 (11.1 %)	2.44 (0.56–10.62)
>7	13 (39.4 %)	20 (60.6 %)		25 (75.8 %)	8 (24.2 %)	**3.56 (1.50–8.44)**	31 (93.9 %)	1 (6.1 %)	1.93 (0.41–9.05)
Handicapping complication									
No	609 (69.0 %)	274 (31.0 %)	1	820 (92.8 %)	64 (7.2 %)	1	782 (88.9 %)	17 (1.9 %)	1
Yes	14 (38.9 %)	22 (61.1 %)	**3.49 (1.76–6.92)**	28 (77.8 %)	8 (22.2 %)	**3.66 (1.60–8.36)**	27 (75.0 %)	9 (25.0 %)	**2.66 (1.21–5.82)**
Health Insurance									
Yes	511 (69.7 %)	222 (30.3 %)	1	679 (92.6 %)	54 (7.4 %)	1	650 (89.2 %)	79 (10.8 %)	1
No	112 (60.2 %)	74 (39.8 %)	**1.52 (1.09–2.12)**	168 (90.4 %)	18 (9.6 %)	1.33 (0.76–2.34)	159 (85.0 %)	28 (15.0 %)	1.44 (0.91–2.30)
Women’s education									
Completed secondary level and reached university level	141 (65.3 %)	75 (34.7 %)	1	194 (89.8 %)	22 (10.2 %)	1	195 (91.1 %)	19 (8.9 %)	1
Completed primary level	411 (67.8 %)	195 (32.2 %)	1.29 (0.80–2.09)	565 (93.1 %)	42 (6.9 %)	0.82 (0.37–1.81)	527 (87.1 %)	78 (12.9 %)	1.28 (0.64–2.58)
No education	71 (73.2 %)	26 (26.8 %)	1.45 (0.85–2.46)	89 (91.8 %)	8 (8.2 %)	1.26 (0.54–2.94)	87 (89.7 %)	10 (10.3 %)	0.84 (0.37–1.89)
Woman occupation									
Employed	437 (67.3 %)	212 (32.7 %)	1	598 (92.0 %)	52 (8.0 %)	1	575 (88.6 %)	74 (11.4 %)	1
Non employed	176 (68.5 %)	81 (31.5 %)	1.05 (0.77–1.43)	238 (92.6 %)	19 (7.4 %)	1.08 (0.63–1.88)	223 (87.8 %)	31 (12.2 %)	0.92 (0.59–1.44)
Infection during pregnancy									
No	610 (67.8 %)	290 (32.2 %)	1	836 (92.8 %)	65 (7.2 %)	1	797 (88.9 %)	100 (11.1 %)	1
Yes	13 (68.4 %)	6 (31.6 %)	0.97 (0.36–2.58)	12 (63.2 %)	7 (36.8 %)	**7.50 (2.85–19.70)**	12 (63.2 %)	7 (36.8 %)	**4.64 (1.78–12.08)**
Alcohol									
No	450 (66.8 %)	224 (33.2 %)	1	616 (91.3 %)	59 (8.7 %)	1	589 (87.5 %)	84 (12.5 %)	1
Yes	172 (70.5 %)	72 (29.5 %)	1.18 (0.86–1.63)	231 (94.7 %)	13 (5.3 %)	1.70 (0.91–3.16)	220 (90.5 %)	23 (9.5 %)	1.36 (0.83–2.21)
Smoking									
No	605 (67.5 %)	291 (32.5 %)	1	826 (92.1 %)	71 (7.9 %)	1	790 (88.5 %)	103 (11.5 %)	1
Yes	18 (78.3 %)	5 (21.7 %)	0.57 (0.21–1.57)	22 (95.7 %)	1 (4.3 %)	0.52 (0.07–3.98)	19 (82.6 %)	4 (17.4 %)	1.61 (0.53–4.83)
Severe bleeding during pregnancy and labour									
No	606 (68.5 %)	279 (31.5 %)	1	821 (92.7 %)	65 (7.3 %)	1	780 (88.3 %)	103 (11.7 %)	1
Yes	17 (50.0 %)	17 (50.0 %)	**2.17 (1.09–4.31)**	27 (79.4 %)	7 (20.6 %)	**3.27 (1.37–7.80)**	29 (87.9 %)	4 (12.1 %)	1.04 (0.36–3.03)
Significant blood loss after delivery									
No	576 (69.6 %)	252 (30.4 %)	1	772 (93.1 %)	57 (6.9 %)	1	737 (89.3 %)	88 (10.7 %)	1
Yes	47 (51.6 %)	44 (48.4 %)	**2.14 (1.38–3.31)**	76 (83.5 %)	15 (16.5 %)	**2.67 (1.44–4.94)**	72 (79.1 %)	19 (20.9 %)	**2.21 (1.27–3.83)**
Breast feeding									
Yes	NA^f^			837 (92.3 %)	70 (7.7 %)	1	798 (88.4 %)	105 (11.6 %)	1
No				4 (66.7 %)	2 (33.3 %)	**5.97 (1.07–33.21)**	6 (100.0 %)	0 (0.0 %)	000000
Household income per month									
> 35,000 RWF	253 (69.7 %)	110 (30.3 %)	1	339 (93.4 %)	24 (6.6 %)	1	333 (91.5 %)	31 (8.5 %)	1
17,500–35,000 RWF	164 (68.6 %)	75 (31.4 %)	1.05 (0.73–1.49)	219 (91.3 %)	21 (8.8 %)	1.35 (0.73–2.49)	208 (87.5 %)	30 (12.6 %)	1.54 (0.91–2.63)
<17,500 RWF	169 (65.5 %)	89 (34.5 %)	1.21 (0.86–1.70)	237 (91.9 %)	21 (8.1 %)	1.25 (0.68–2.30)	218 (85.5 %)	37 (14.5 %)	**1.82 (1.09–3.02)**

^a^Good-SRH (Self-rated health status) includes very good and good health status categories

^b^Poor-SRH (Self-rated health status) includes very poor and poor health status categories

^c^Univariable logistic regression analysis with calculation of crude odds ratio (OR) and its 95 % confidence interval (CI)

^d^Three times post partum: one day, one month after delivery, and at the time of the interview

^e^Statistically significant odds ratios are in bold

^f^NA: Not applicable

**Table 4:** Univariable and multivariable and logistic regression analysis^a^ of poor-SRH^b^ at three times post partum^c^

	Poor-SRH^b^ at day one after delivery		Poor-SRH at one month after delivery		Poor-SRH at the time of the interview	
	Univariable analysis	Multivariable analysis	Univariable analysis	Multivariable analysis	Univariable analysis	Multivariable analysis
	Crude Odds Ratios and its 95 % CI	Adjusted^d^ Odds Ratios and its 95 % CI	Crude Odds Ratios and its 95 % CI	Adjusted^d^ Odds Ratios and its 95 % CI	Crude Odds Ratios and its 95 % CI	Adjusted^d^ Odds Ratios and its 95 % CI
Women’s age (years)						
< 25	1.02 (0.75–1.38)	1.07 (0.72–1.60)	**1.93 (1.12–3.32)** ^**e**^	**2.71 (1.30–5.62)**	0.82 (0.51–1.31)	0.87 (0.46–1.64)
25–34	1	1	1	1	1	1
> 35	0.86 (0.57–1.31)	0.94 (0.56–1.58)	1.95 (0.99–3.85)	1.44 (0.61–3.40)	1.10 (0.62–1.94)	0.77 (0.37–1.57)
Place of delivery						
At health facility	1		1		1	1
At home or on the way to health facility	0.75 (0.38–1.48)		1.83 (0.74–4.47)		**2.48 (1.22–5.04)**	1.92 (0.87–4.21)
Mode of delivery						
Vaginal (with or without instruments)	1	1	1	1	1	1
Caesarean Section	**3.48 (2.31–5.23)**	**3.20 (2.07–4.96)**	**2.28 (1.25–4.14)**	2.25 (0.97–5.23)	0.62 (0.30–1.28)	0.66 (0.29–1.49)
Hypertension during pregnancy and delivery						
No	1	1	1	1	1	
Yes	**2.99 (1.67–5.37)**	**2.38 (1.22–4.62)**	**2.88 (1.34–6.21)**	1.30 (0.47–3.55)	1.27 (0.55–2.92)	
Convulsions during pregnancy, delivery and post partum						
No	1		1	1	1	
Yes	1.81 (0.92–3.58)		**2.56 (1.02–6.40)**	0.76 (0.21–2.67)	2.02 (0.86–4.77)	
Diabetes mellitus during pregnancy						
No	1		1		1	
Yes	0.69 (0.25–1.93)		0.000		0.83 (0.19–3.65)	
Anemia during pregnancy						
No	1	1	1	1	1	1
Yes	**1.59 (1.10–2.31**)	1.34 (0.88–2.01)	**2.19 (1.25–3.84)**	**2.37 (1.25–4.49)**	**4.33 (2.77–6.75)**	**4.45 (2.69–7.35)**
Marital status						
Married or cohabiting	1	1	1	1	1	1
Unmarried or single or widow or separated	**1.42 (0.95–2.12)**	**1.60 (1.02–2.51)**	1.05 (0.53–2.05)	1.51 (0.66–3.47)	**0.60 (0.37–0.99)**	**0.49 (0.26–0.93)**
Age at marriage (years)						
< 20	1		1		1	
≥ 20	1.21 (0.87–1.67)		1.25 (0.70–2.22)		0.81 (0.50–1.26)	
Number of births						
1	1	1	1	1	1	1
2–4	1.14 (0.82–1.59)	1.19 (0.80–1.76)	1.10 (0.61–1.96)	1.55 (0.74–3.22)	1.38 (0.82–2.32)	1.71 (0.89–3.29**)**
> 4	0.72 (0.48–1.07)	0.76 (0.44–1.61)	1.15 (0.59–2.24)	2.26 (0.80–6.35)	**1.87 (1.07–3.27)**	**2.42 (1.06–5.54)**
Time when discharged (days)						
< 3	NA^f^		**1**	1	1	
3			**0.51 (0.26–0.99)**	**0.49 (0.24–0.99)**	1.83 (0.42–7.90)	
4–7			**1.02 (0.49–2.11)**	0.64 (0.25–1.64)	2.44 (0.56–10.62)	
> 7			**3.56 (1.50–8.44)**	2.47 (0.83–7.30)	1.93 (0.41–9.05)	
Handicapping complication						
No	1	1	1	1	1	1
Yes	**3.49 (1.76–6.92)**	2.16 (0.99–4.69)	**3.66 (1.60–8.36)**	2.23 (0.80–6.23)	**2.66 (1.21–5.82)**	2.20 (0.89–5.47)
Health Insurance						
Yes	1	1	1		1	
No	**1.52 (1.09–2.12)**	**1.59 (1.11–2.28)**	1.33 (0.76–2.34)		1.44 (0.91–2.30)	
Women’s education						
Completed secondary level or reached university level	1		1		1	
Completed primary level	1.29 (0.80–2.09)		0.82 (0.37–1.81)		1.28 (0.64–2.58)	
No education	1.45 (0.85–2.46)		1.26 (0.54–2.94)		0.84 (0.37–1.89)	
Woman occupation						
Employed	1		1		1	
Non employed	1.05 (0.77–1.43)		1.08 (0.63–1.88)		0.92 (0.59–1.44)	
Infection during pregnancy						
No	1		1	1	1	1
Yes	0.97 (0.36–2.58)		**7.50 (2.85–19.70)**	**6.94 (2.14–22.53)**	**4.64 (1.78–12.08)**	**3.73 (1.20–11.59)**
Alcohol						
No	1		1		1	
Yes	1.18 (0.86–1.63)		1.70 (0.91–3.16)		1.36 (0.83–2.21)	
Smoking						
No	1		1		1	
Yes	0.57 (0.21–1.57)		0.52 (0.07–3.98)		1.61 (0.53–4.83)	
Severe bleeding during pregnancy and labour						
No	1	1	1	1	1	
Yes	**2.17 (1.09–4.31)**	1.74 (0.77–3.92)	**3.27 (1.37–7.80)**	**2.96 (1.09–8.02)**	1.04 (0.36–3.03)	
Significant blood loss after delivery						
No	1	1	1	1	1	1
Yes	**2.14 (1.38–3.31)**	**2.04 (1.24–3.35)**	**2.67 (1.44–4.94)**	1.77 (0.81–3.82)	**2.21 (1.27–3.83)**	1.22 (0.60–2.45)
Breast feeding						
Yes	NA^f^		1	1	1	
No			**5.97 (1.07–33.21)**	**9.54 (1.50–60.39)**	0	
Household income per month						
> 35,000 RWF	1		1		1	1
17,500–35,000 RWF	1.05 (0.73–1.49)		1.35 (0.73–2.49)		1.54 (0.91–2.63)	1.47 (0.83–2.62)
< 17,500 RWF	1.21 (0.86–1.70)		1.25 (0.68–2.30)		**1.82 (1.09–3.02)**	1.65 (0.94–2.87)

^a^Univariable logistic regression analysis with calculation of crude odds ratio (OR) and its 95 % confidence interval (CI)

^b^Poor-SRH (Poor self-rated health status) includes very poor and poor health status categories and Good-SRH (Good self-rated health status) includes very good and good health status categories

^c^Three post partum times: one day, one month after delivery, and at the time of the interview

^d^Adjusted Odds Ratios for women’s age, number of births, mode of delivery, and marital status

^e^Statistically significant odds ratios are in bold

^f^NA: Not applicable

#### Self-rated overall health status at one week postpartum

The univariable logistic regression analyses revealed several variables statistically significally associated with poor-SRH one week postpartum: anaemia during pregnancy (OR = 1.78, CI = 1.15–2.75; ref.: no anaemia), caesarean section as mode of delivery (OR = 2.63, CI = 1.68–4.11; ref.: vaginal delivery with or without instruments), hypertension during pregnancy and delivery (OR = 3.41, CI = 1.87–6.24; ref.: no hypertension), severe bleeding during pregnancy and labour (OR = 3.69, CI = 1.82–7.48; ref.: no severe bleeding), significant postpartum haemorrage (OR = 2.60, CI = 1.61–4.21; ref.: no significant postpartum haemorrage), handicapping complication (i.e., postpartum fistula or fecal and urinary incontinance) (OR = 3.34, CI = 1.67–6.70; ref.: no handicapping complication), woman’s age less than 25 year (OR = 1.61, CI = 1.10–2.35; ref.: age group 25 to 34 years of age), discharge time seven days postpartum (OR = 4.32, CI = 2.07–9.02; ref.: discharge time less than 3 days), and convulsions during pregnancy, delivery, and up to one month postpartum (OR = 2.35, CI = 1.12–4.91; ref.: no convulsions).

In a multivariable logistic regression model (*n* = 858), the following reproductive factors were statistically significantly associated with poor-SRH one week postpartum: caesarean section (OR = 1.95, CI = 1.08–5.53), severe bleeding during pregnancy and labour (OR = 3.60, CI = 1.56–8.31), hypertension during pregnancy and delivery (OR = 2.21, CI = 1.06–4.60), significant postpartum haemorrage (OR = 2.01, CI = 1.12–3.58), woman’s age less than 25 years (OR = 1.71, CI = 1.05–2.80), and discharge time more than seven days postpartum (OR = 2.81, CI = 1.18–6.66).

#### Self-rated overall health status at one month postpartum

In the univariable logistic regression, anaemia and infection during pregnancy, caesarean section, severe bleeding during pregnancy and labour, significant postpartum haemorrage, and handicapping complication (i.e., postpartum fistula or fecal and urinary incontinance) were associated with poor-SRH at one month postpartum (Table 3). In the multivariable logistic regression model, anaemia and infection during pregnancy, breastfeeding, severe bleeding during pregnancy and labour, and age less than 25 years were significantly associated with poor-SRH. Being discharged at third day postpartum was protective for reporting poor health at one month postpartum (OR = 0.49, CI = 0.24–0.99; ref.: discharge time before three days postpartum) (Table 4).

#### Self-rated overall health status at time of interview

For SRH at time of interview, the univariable logistic regression revealed an association with poor-SRH and place of delivery (i.e., at home or on the way to the health facility), anaemia and infection during pregnancy, handicapping complication (i.e., postpartum fistula or fecal and urinary incontinence), multiparity, and marital status (Table 3). In the multivariable analysis, multiparity was associated with poor-SRH at the time of interview (Table 4).

#### Poor self-rated overall health at several times

For SRH at one day and one week postpartum, the multivariable logistic regression model revealed an association with poor-SRH and caesarean section, hypertension during pregnancy and delivery, and significant postpartum haemorrage. Severe bleeding during pregnancy and labour was associated with poor-SRH at one week and one month postpartum. Infection and anaemia during pregnancy were associated with poor-SRH at one month postpartum (OR = 7.15, CI = 2.17–23.50 and OR = 2.32, CI = 1.22–4.38, respectively) and at the time of the interview (OR = 3.36, CI = 1.17–11.56) and OR = 4.48, CI = 2.78–7.21, respectively) (Table 4).

## Discussion

This study found that approximately one-third of delivered women reported poor-SRH at one day postpartum. Participants’ overall health status generally improved during the following year postpartum. Background factors such as marital status, age at marriage, education, occupation, smoking, and use of alcohol were not determinants of poor-SRH during the whole postpartum period. This study estimated the prevalence of poor-SRH to be 32.2 % at one day, 7.8 % at one month, and 11.2 % at the time of the interview. The most plausible explanation of the increase of poor-SRH from one month postpartum to the time of interview is that women may have developed new health problems during their postpartum year. It is difficult to compare findings of this study with previous studies that have estimated poor health status at different times postpartum, as they have reported prevalences in the range of 4 % to 15 % between one and two years postpartum [18, 27, 28]. Nevertheless, we consider our poor-SRH prevalence at one day as comparatively high in the light of these previous studies.

Hypertension during pregnancy and delivery and significant postpartum haemorrhage were associated with poor-SRH at one day and one week postpartum, and severe bleeding during pregnancy and delivery were associated with poor-SRH at one week and one month postpartum. These findings are consistent with the literature that have identified these factors to be important determinants of poor-SRH during the early postpartum period [27, 29, 30].

There are differing results on the impact of caesarian section on poor health status postpartum; in some studies poor health status at early postpartum is associated with caesarean section, [10, 31], whereas others have not found any association [27, 32]. Our study showed caesarean section to be associated with poor-SRH at one day and one week after the operation.

The only sociodemographic factor that was associated with poor-SRH at one week and one month postpartum was age less than 25 years in relation to age 25 to 34 years. Similar results have also been shown in other studies [5, 33]; these results might be explained by the fact that young mothers are often more emotionally and physically vulnerable [5]. Low level of education was not found to be associated with poor-SRH in our study although other studies report it as a predictor of poor health status postpartum [5, 10]. In Rwanda, utilization rate of maternal services before and during delivery is very high [23]. Both women with low and high levels of education have the same access to maternal health services including CHWs’ support in the community, thus pregnant women’s access is the same irrespective of educational levels [24, 34].

In this study, multiparity was associated with poor-SRH at the time of the interview, a finding consistent with other studies that report multiparity to be associated with postpartum poor health status [15, 17]. Multiparity constitutes the potential risks of pregnancy and postpartum adverse outcomes [15].

Rwandan women who have experienced an uncomplicated delivery at a health facility are commonly discharged one to three days postpartum [23]. In our study, discharge on the third day postpartum in relation to discharge before three days was found to be protective of poor-SRH at one month postpartum. These results are coherent with the WHO recommendations to discharge women three days after giving birth [35]. Facility-based health care provided during the early postpartum period may have a positive effect on women’s overall health status during the whole postpartum period.

Previous studies performed in low and middle income countries have found pregnancy-related factors such as anaemia and infection during pregnancy associated with health problems several months postpartum [27, 29]. In our study, the main determinants of postpartum poor-SRH after one month were anaemia and infection during pregnancy.

### Methodological considerations

One strength of this study is that almost all households and women selected were reached and consented to participate in the study. We consider the sample to be representative of the population under study. Another strength of this study was that female nurses, midwives, and clinical psychologists were used as interviewers in order to make female participants confident enough to respond. The representativity of this study undertaken in the Northern Province and Kigali is also supported by the fact that the data obtained for prevalence rates of ANC use, delivery at health facility, caesarean section, and postpartum care attendance were very similar to data available on maternal services delivery and use in Rwanda in the latest published Demographic Health Survey (DHS) 2014–15 results [23]. Recall bias may have been an issue since data were collected retrospectively, although previous studies have shown that recall bias is not a major issue when women are recalling their reproductive history [36, 37]. Another issue is that there may be underreporting or overreporting of some health conditions. In this study, the average time between the date of the interview and the date of the latest delivery was 7.1 months with a range of 1.4-14.3 months. This means that the time of interview for all participants corresponds to a wide time interval. Therefore, the results are influenced by the fact that some participants were interviewed at an early time point during the postpartum period when the health problems were still prevalent, whereas other participants were interviewed at a later time point when their health problems could have been resolved.

## Conclusions

This study reports a high prevalence of poor SRH among Rwandan women in the early postpartum period. Infection, anaemia, hypertension, and severe bleeding during pregnancy and significant postpartum haemorrhage were the main determinants of poor-SRH during postpartum. Discharge time at third day postpartum in relation to discharge before three days postpartum decreased the risk of reporting poor-SRH at a later time. These incriminated determinants of poor-SRH are pregnancy and delivery-related factors, factors that may be prevented or reduced by more frequent and particular attention during pregnancy. Timely measures should be undertaken to deal with complications during delivery and early postpartum. The simple SRH status measurement can be used to identify determinants of poor health status. Further research is warranted to determine whether SRH inquiry may be used during antenatal care and postnatal care visits to screen for health problems and pregnancy complications related to poor health status postpartum.
